# T-cell responses to SARS-CoV-2 in unexposed South African women

**DOI:** 10.12688/gatesopenres.13373.2

**Published:** 2022-07-13

**Authors:** Marta C. Nunes, Michael J. Johnson, Gaurav Kwatra, Adriana Weinberg, Shabir A. Madhi

**Affiliations:** 1 South African Medical Research Council, Vaccines and Infectious Diseases Analytics Research Unit, School of Pathology, Faculty of Health Sciences, University of the Witwatersrand, Johannesburg, South Africa; 2 Department of Science and Technology/National Research Foundation, South African Research Chair Initiative in Vaccine Preventable Diseases, Faculty of Health Sciences, University of the Witwatersrand, Johannesburg, South Africa; 3 Department of Pediatrics, Medicine and Pathology, University of Colorado, Anschutz Medical Campus, Aurora, Colorado, USA

**Keywords:** cell mediated immunity, SARS-CoV-2, interferon gamma, interleukin 2

## Abstract

**Background**: A potential explanation for the fact that the high rate of infection of SARS-CoV-2 in South Africa did not translate into high rates of severe illness and death may be the presence of cross-reactive immunity induced by common cold coronaviruses (CCoV).

**Methods**: We used SARS-CoV-2 peptide pools and whole virus antigen to stimulate peripheral blood mononuclear cells collected pre-2020 from South African women. Dual-colour FluoroSpot assay was used to measure interferon gamma (IFNγ) and interleukin 2 (IL2) production.

**Results**: Among the 97 study participants, IFNγ responses were observed in 29.9% of the women and IL2 among 39.2%. Overall, 51.6% of women demonstrated response to at least one stimulant.

**Conclusion**: We demonstrate the presence of cross-reactive immunity to SARS-CoV-2, which might have been induced by past exposure to CCoV.

## Introduction

South Africa experienced a higher rate of SARS-CoV-2 infection (approximately 40% based on convenience sampling sero-survey in one area) during the course of the first COVID-19 wave compared with the global North (highest estimates of 11% in Italy and 13% in the USA) ^1– 3^. Nevertheless, the COVID-19 mortality rate in South Africa (284 per 1,000,000) was lower than that observed in high-income countries such as in Spain, Italy, USA and United Kingdom (594-684 per 1,000,000) ^4^. Possible reasons for the lower risk of progression of SARS-CoV-2 infection to severe COVID-19 in low–and-middle income settings compared to high-income settings include differences in age-group demographics, prevalence of underlying co-morbidities, genetic factors and factors that could influence the virus inoculum load. Other possible reasons include underpinning cross-reactive cellular immunity that mitigated progression of SARS-CoV-2 infection to COVID-19 severe disease and death. Previous studies demonstrated T-cell responses against SARS-CoV-2 in uninfected individuals and postulated that immunity induced by human endemic common cold coronaviruses (CCoV) may confer cross-reactive immune responses ^5^. Underlying anamnestic cell mediated immunity, whilst not necessarily able to prevent infection with SARS-CoV-2, might attenuate the clinical course of illness and prevent progression to severe COVID-19 ^6^. Due to high population density and overcrowding, exposure to CCoV might be more intense in African than in higher-income settings, as is the case for other respiratory pathogens ^7^. In the international case-control study Pneumonia Etiology Research for Child Health (PERCH), 25% of healthy children in Soweto, South Africa were found to be colonized with CCoV ^8^. The frequent exposure of the adult contacts to CCoV is likely to boost their immune responses to CCoV.

To inform whether cross-reactive immunity might have contributed to the COVID-19 epidemiological experience in South Africa, we investigated cellular immunity to SARS-CoV-2 in samples collected in the pre COVID-19 pandemic era.

## Methods

### Ethics statement

The study was approved by the Human Research Ethics Committee of the University of the Witwatersrand (201128) and done in accordance with Good Clinical Practice guidelines. Written informed consent was obtained from the South African participants when they were enrolled into the original studies, including consenting for future use of their samples. For the USA samples no additional ethics committee approvals were required per NIH/COMIRB definition of human subject studies.

### Samples

Peripheral blood mononuclear cells (PBMCs) collected under studies conducted at the Vaccines and Infectious Diseases Analytics (VIDA) research unit during 2013 and 2017 were analysed ^9^. The sample set included cells from South African pregnant or post-partum women, living with and without HIV who participated in an influenza vaccine trial during 2013 ^9^ or who were enrolled at delivery into a longitudinal study in 2017. The PBMCs used were a convenience sample of available leftover cells. PBMCs were initially separated from blood by Ficoll-Hypaque density gradient centrifugation (Sigma Diagnostics), cryopreserved and stored in liquid nitrogen according to standardized protocols and were shipped, also in liquid nitrogen containers, to the University of Colorado, USA ^10^. Cells were thawed slowly as previously described ^11^. Leukopaks were obtained from COVID-19 convalescent non-pregnant individuals without HIV at Children’s Hospital Colorado Blood Donor Center, USA. PBMCs were separated as described above and used as positive controls.

### Laboratory procedures

Cryopreserved PBMCs were thawed as previously described ^11^. Following overnight rest, PBMCs were stimulated for 48 hours in 96-well dual-colour interferon gamma (IFNγ) and interleukin 2 (IL2) FluoroSpot plates (Mabtech catalog number FSP-0102-10; capture antibodies: monoclonal antibodies 1-D1K and MT2A91/2C95; detection antibodies: BAM-conjugated monoclonal antibody 7-B6-1 and biotinylated monoclonal antibody MT8G10) with pre-optimized amounts of SARS-CoV-2 irradiated cell lysate, 1μg/ml spike (S) protein peptides megapool (pool of peptides spanning the entire sequences of the S protein, courtesy of Dr Weiskopf from La Jolla Institute [LJI]), 1μg/ml non-S peptides megapool (predicted epitopes from the non-S region of the viral genome, LJI), 2μg/ml CD8 peptide megapool A (LJI), or CD8 peptide megapool B (CD8-A and CD8-B peptides collectively cover 628 predicted HLA class I CD8+ T-cell epitopes from the entire SARS-CoV-2 proteome, with CD8-A megapool containing S epitopes, among epitopes to other proteins, LJI) in duplicate wells at 250,000 cells/well ^5, 12^. Unstimulated negative and phytohemagglutinin (PHA, Sigma) positive controls were included. Bound cytokines were revealed as per the manufacturer’s instructions and read using an Immunospot II instrument (Cellular Technology Limited.).

### Analysis

Results were expressed as spot-forming-cells (SFC)/10 ^6^ PBMC in antigen- or mitogen-stimulated wells after subtraction of SFC in the unstimulated control wells.

Demographic characteristics of the South African women were described as percentages or means with standard deviations (SD). Geometric mean number of SFC/10 ^6^ PBMCs and the corresponding 95% confidence interval (95%CI) were estimated using logarithmic transformation and compared between study cohorts by Student’s t-test. Responders were defined as individuals with ≥20 SFCs/10 ^6^ PBMCs after subtraction of the SFCs in unstimulated control wells and with concomitant ≥2-fold increase over the unstimulated wells, and the proportion of responders were compared by Chi-square or Fisher’s exact-tests.

Analyses were performed using STATA version 13.1 (College Station, TX, USA).

An earlier version of this article can be found on Research Square (doi: https://doi.org/10.21203/rs.3.rs-471880/v1).

## Results

Peripheral blood mononuclear cells from 97 South African women were analysed ^13^. This included 33 pregnant and 10 non-pregnant women living with HIV, 38 pregnant and 16 non-pregnant women without HIV ( Table 1). PBMCs from seven convalescent individuals diagnosed with COVID-19 were included as controls and comparators.

**Table 1.** Characteristics of the South African women participating in the study.

	N=97
2013 enrolments	55 (56.7)
2017 enrolments	42 (43.3)
Mean age (SD), years	27.3 (6.0)
Living with HIV	43 (44.3)
CD4+ cell count ≥350 cells/ml	17 (42.5) [40]
HIV viral load <40 copies/ml	15 (42.9) [35]
On antiretroviral therapy	38 (88.4)
Pregnant	71 (73.2)
Women in the second trimester	23 (32.4)
Women in the third trimester	48 (67.6)

Results are n (%) unless stated otherwise. Numbers in square brackets represent the number of participants with available information. SD: standard deviation.

Table 2 summarizes the responses, and shows that overall, IFNγ responses were detected in 6.2% after stimulation with each spike or non-spike pool in South African women. CD8+ T-cell responses were detected in 5.2% of the women using CD8-A pool and 20.6% after CD8-B pool stimulation. Responses were, however, observed in just 1% after stimulation with SARS-CoV-2 irradiated cell lysate. Non-pregnant women showed better response (in terms of SFC geometric mean and percentage of responders) compared to pregnant women after spike stimulation (15.4% vs. 2.8%, p=0.043; respectively). A higher percentage of women without HIV (11.1%) also had responses compared with women living with HIV (0%, p=0.032) after spike stimulation. Overall, 29.9% of women demonstrated response to at least one stimulant. IFNγ responses were evident in all seven convalescent 2020 samples across stimulants, except for CD8-B with only 28.6% showing a response.

**Table 2.** Interferon γ and Interleukin 2 responses assessed as number of spot-forming-cells or percentage of responders among study participants after stimulation with SARS-CoV-2 peptide pools and whole virus.

	Spike	Non-spike	CD8-A	CD8-B	Irradiated cell lysate	At least one response
**Interferon γ**						
**SFCs per 10 ^6^ PBMCs geometric mean (95%CI)**						
Overall pre-2020 participants	8.0 (6.2, 10.3)	8.3 (6.8, 10.0)	7.6 (5.5, 10.4)	21.2 (13.1, 34.3)	5.3 (4.0, 7.1)	
Pregnant women	6.2 (4.8, 8.0) ^a^	8.5 (6.9, 10.5)	6.9 (5.2, 9.3)	20.0 (11.5, 34.8)	5.4 (3.8, 7.5)	
Non-pregnant women	13.9 (8.2, 23.5)	7.8 (4.9, 12.3)	9.1 (4.0, 20.7)	23.6 (8.8, 63.6)	5.1 (2.5, 10.3)	
Women living with HIV	6.3 (4.5, 8.8)	7.9 (6.0, 10.3)	7.8 (4.6, 13.1)	21.8 (10.1, 46.9)	5.8 (3.6, 9.3)	
Women without HIV	9.6 (6.7, 13.8)	8.6 (6.5, 11.4)	7.4 (4.9, 11.2)	20.7 (10.8, 39.8)	5.0 (3.4, 7.4)	
2020 participants	309.3 (153.1, 624.7)	99.8 (52.2, 190.7)	124.8 (59.4, 262.0)	10.0 (3.8, 25.9)	97.9 (48.1, 199.0)	
**Responders (%)**						
Overall pre-2020 participants	6 (6.2)	6 (6.2)	5 (5.2)	20 (20.6)	1 (1.0)	29 (29.9)
Pregnant women	2 (2.8) ^a^	4 (5.6)	2 (2.8)	14 (19.7)	1 (1.4)	19 (26.8)
Non-pregnant women	4 (15.4)	2 (7.8)	3 (11.5)	6 (23.1)	0	10 (38.5)
Women living with HIV	0 ^b^	2 (4.7)	2 (4.7)	7 (16.3)	1 (2.3)	11 (25.6)
Women without HIV	6 (11.1)	4 (7.4)	3 (5.6)	13 (24.1)	0	18 (33.3)
2020 participants	7 (100)	7 (100)	7 (100)	2 (28.6)	7 (100)	7 (100)
**Interleukin 2**						
**SFCs per 10 ^6^ PBMCs geometric mean (95%CI)**						
Overall pre-2020 participants	10.0 (7.9, 12.6)	12.6 (10.5, 15.2)	6.7 (5.2, 8.6)	9.4 (7.1, 12.4)	8.4 (6.5, 10.9)	
Pregnant women	8.4 (6.4, 11.0) ^a^	13.7 (11.0, 17)	6.6 (5.0, 8.7)	9.7 (7.0, 13.4)	8.0 (5.9, 10.9)	
Non-pregnant women	15.6 (10.1, 24.0)	10.5 (7.3, 15.1)	7.0 (3.6, 13.6)	8.5 (4.6, 15.9)	9.7 (5.6, 16.9)	
Women living with HIV	8.9 (6.0, 13.3)	10.8 (7.7, 14.9)	6.4 (4.1, 10)	8.9 (5.8, 13.8)	8.3 (5.5, 12.4)	
Women without HIV	10.6 (7.9, 14.4)	14.1 (11.3, 17.5)	6.9 (5.0, 9.5)	9.7 (6.6, 14.2)	8.5 (6.0, 12.3)	
2020 participants	344.0 (195.5, 605.3)	177.8 (103.8, 304.7)	55.3 (30.9, 98.9)	16.1 (8.9, 29.1)	153.4 (81.7, 288.4)	
**Responders (%)**						
Overall pre-2020 participants	15 (15.5)	22 (22.7)	6 (6.2)	12 (12.4)	3 (3.1)	38 (39.2)
Pregnant women	9 (12.7)	18 (25.4)	5 (7.0)	10 (14.1)	3 (4.2)	29 (40.9)
Non-pregnant women	6 (23.1)	4 (15.4)	1 (3.9)	2 (7.7)	0	9 (34.6)
Women living with HIV	5 (11.6)	8 (18.6)	3 (7.0)	4 (9.3)	1 (2.3)	11 (25.6) ^b^
Women without HIV	10 (18.5)	14 (25.9)	3 (5.6)	8 (14.8)	2 (3.7)	27 (50.0)
2020 participants	7 (100)	7 (100)	6 (85.7)	1 (14.3)	7 (100)	7 (100)
**Responders (%) to either Interferon g or Interleukin 2**						
Overall pre-2020 participants	17 (17.5)	24 (24.7)	10 (10.3)	25 (25.8)	4 (4.1)	50 (51.6)
Pregnant women	10 (14.1)	20 (28.2)	7 (9.9)	19 (26.8)	4 (5.6)	37 (52.1)
Non-pregnant women	7 (26.9)	4 (15.4)	3 (11.5)	6 (23.1)	0	13 (50.0)
Women living with HIV	5 (11.6)	9 (20.9)	5 (11.6)	9 (20.9)	2 (4.7)	17 (39.5) ^b^
Women without HIV	12 (22.2)	15 (27.8)	5 (9.3)	16 (29.6)	2 (3.7)	33 (61.1)
2020 participants	7 (100)	7 (100)	7 (100)	2 (28.6)	7 (100)	7 (100)

Responders are women with ≥20 SFCs after subtracting media control and with concomitant ≥2-fold increase from media only stimulation. SFCs: Spot forming cells. 95%CI: 95% confidence interval. ^a^p-value < 0.05 pregnant vs. non-pregnant. ^b^p-value < 0.05 living with HIV vs. without HIV.

Interleukin 2 was produced in response to spike and non-spike pools by 15.5% and 22.7% of the South African women, respectively. CD8+ T-cell responses were detected in 6.2% and 12.4% of the women after CD8-A and CD8-B pools stimulation, respectively. SARS-CoV-2 irradiated cell lysate elicited responses in 6.2% of women. Non-pregnant women had significantly higher SFC geometric mean compared to pregnant women after spike stimulation (p=0.017). Overall, 39.2% of women demonstrated response to at least one stimulant, with this being higher in women without HIV (50%) than in women living with HIV (25.6%, p=0.014). All seven convalescent 2020 patients demonstrated IL2 responses to at least one stimulant, however, only one (14.3%) participant showed response after CD8-B pool incubation.

Considering either IFNγ or IL2 production, 51.6% of women demonstrated response to at least one stimulant. Women without HIV (61.1%) demonstrated better overall response than women living with HIV (39.5%, p=0.035).

## Discussion

Using PBMCs collected before 2020, in this antigen-specific analysis we confirmed that approximately 50% of adult South African women, who had not been exposed to SARS-CoV-2, had cellular immune responses against peptides derived from SARS-CoV-2. This is similar to the frequency reported in studies from the USA (40–60%), Singapore (51%) and Europe (35%) ^12, 14, 15^. Notably, adult plasma samples collected prior to 2020 from a similar cohort in South Africa as used in this study showed no reactivity to the receptor binding domain of the immunogenic SARS-CoV-2 spike protein when tested by an in-house Luminex assay ^16^.

Since CCoV and SARS-CoV-2 belong to the same coronavirinae subfamily, pre-existing CCoV-specific T cells could recognize SARS-CoV-2, and lead to SARS-CoV-2 reactive cells in unexposed individuals. In theory, cross-reactive cells in SARS-CoV-2 naïve individuals could mount a rapid adaptive immune response against the novel virus and protect them from infection ^17^ or impact the clinical outcomes of the disease, as suggested by a study finding that recent CCoV infections were associated with less severe COVID-19 ^18^.

The differential magnitude of response elicited by CD8-A and CD8-B pools in convalescent individuals in our study has been noted before and may be related to the fact that the CD8-A pool contains immunodominant spike epitopes and other structural proteins ^12^. Notably, in SARS-CoV-2 naive individuals the IFNγ response to CD8-B pool was higher than to any of the other stimulants, suggesting highest cross reactivity between CCoV and SARS-CoV-2 at the level of CD8 T-cell epitopes in non-structural proteins ^17, 19^. These findings are consistent with the observation that the SARS-CoV-2 nucleocapsid protein may induce an immunodominant response in both COVID-19-recovered individuals and in subjects that have not been exposed to SARS-CoV-2 ^20^. This is relevant for the design of new vaccines that might include non-structural targets combined with the spike protein to maintaining the benefit of vaccination against novel viral that escape naturally acquired or current vaccine-induced humoral immunity.

The IFNγ assay predominantly measures effector responses, while the IL2 mainly measures memory responses. As such, IL2 responses were slightly higher than IFNγ responses to the whole virus inactivated antigen, typically processed and presented in the context of HLA Class II. IL2 production in response to spike and non-spike pools was also higher than IFNγ, consistent with memory CD4 T-cell stimulation. In contrast, the CD8 pools elicited slightly higher IFNγ responses. The higher proportion of SARS-CoV-2 naive women with IL2 production after SARS-CoV-2 antigenic stimulation suggests that memory responses may be more sensitive than effector responses for the detection of SARS-CoV-2 cross-reactive responses generated by past infection with CCoV. Moreover, the majority of PBMCs analysed were collected from pregnant women and it is well established that IFNγ production decreases in pregnancy ^21^.

Although women living with HIV had lower responses compared to women without HIV, cross-reactivity was still detected among women with HIV, which might explain why many reports, albeit not all, did not identify HIV infection as a risk factor for severe COVID-19 ^22, 23^.

While the South African women might have recently received influenza and/or tetanus vaccines, a limitation of our study is that we do not know the bacillus Calmette–Guérin (BCG) vaccination status of the study participants, and we did not account for these heterologous vaccinations, as it has been described that some vaccinations may affect the immune responses to SARS-CoV-2 ^24, 25^. 

In conclusion, in this pilot study we demonstrate the presence of cross-reactive immunity to SARS-CoV-2 among South African women that has possibly been induced by past exposure to CCoV. Whether this immunity is relevant in influencing clinical outcomes still needs to be demonstrated.

## Data availability

### Underlying data

Figshare: pre_covid_Aug2021.csv https://doi.org/10.6084/m9.figshare.16699963.v1 ^13^.

Data are available under the terms of the Creative Commons Attribution 4.0 International license (CC-BY 4.0).
