# Supplementary material for: T-cell responses to SARS-CoV-2 in unexposed South African women
Source: Gates Open Res. 2022 Jul 13;5:150. Originally published 2021 Oct 6. [Version 2] doi: 10.12688/gatesopenres.13373.2 (PMC9279637; doi:10.12688/gatesopenres.13373.2)
Supplement: Peer review history

# Peer review: Reviewer response for version 2

**DOI:** 10.21956/gatesopenres.15006.r32251

**Published:** 2022-07-13

**Authors:** Marcin Ratajewski

**Affiliations:** 1 Institute of Medical Biology, Laboratory of Epigenetics, Polish Academy of Sciences, Łódź, Poland

I have no further comments.

Is the work clearly and accurately presented and does it cite the current literature?

Partly

If applicable, is the statistical analysis and its interpretation appropriate?

Partly

Are all the source data underlying the results available to ensure full reproducibility?

Yes

Is the study design appropriate and is the work technically sound?

Yes

Are the conclusions drawn adequately supported by the results?

Partly

Are sufficient details of methods and analysis provided to allow replication by others?

Partly

Reviewer Expertise:

Immunology

I confirm that I have read this submission and believe that I have an appropriate level of expertise to confirm that it is of an acceptable scientific standard.

# Peer review: Reviewer response for version 1

**DOI:** 10.21956/gatesopenres.14618.r32047

**Published:** 2022-05-25

**Authors:** Marcin Ratajewski

**Affiliations:** 1 Institute of Medical Biology, Laboratory of Epigenetics, Polish Academy of Sciences, Łódź, Poland

Nunes *et al.* show results indicating that PBMCs collected from pre-pandemic donors respond to SARS-CoV-2 viral protein antigens, indicating that a certain pool of the South African population has resistance to this virus. The authors point out that this may be caused by a cross-reactivity induced by infections with the common cold coronaviruses, which previously was suggested by the others.

 Major points:

1. The authors used very high concentrations of peptide pools (1-2 mg/ml) while others used concentrations up to 1μg/ml. Please explain this. Did the authors perform any preliminary experiments to choose the optimal concentrations?
2. Table 2. It is completely incomprehensible, what do the values in the brackets mean e.g. (6.2, 10.3)? The authors should present their results in a comprehensible and clear way. This is not the case here.
3. The discussion is very cursory, and I believe the authors should put more emphasis on it. First, more articles that present data from unexposed donors responding to SARS-CoV-2 proteins should be cited and discussed. The authors should consider the vaccination status of the donors tested, as it is known that not only past infections with common cold coronaviruses but also vaccinations (with e.g. BCG or pneumococcal) can affect the immune system's response to this virus.
4. The authors explain the response of some unexposed donors to SARS-CoV-2 by the cross-reactivity induced by common cold coronavirus infections. It is known that these infections are seasonal. The authors should present what the climate is in South Africa; when it most often comes to common colds; and what the scale is (how many people are infected with common cold coronaviruses).
5. Please reveal statistical tests that have been used to analyze results.

Is the work clearly and accurately presented and does it cite the current literature?

Partly

If applicable, is the statistical analysis and its interpretation appropriate?

Partly

Are all the source data underlying the results available to ensure full reproducibility?

Yes

Is the study design appropriate and is the work technically sound?

Yes

Are the conclusions drawn adequately supported by the results?

Partly

Are sufficient details of methods and analysis provided to allow replication by others?

Partly

Reviewer Expertise:

Immunology

I confirm that I have read this submission and believe that I have an appropriate level of expertise to confirm that it is of an acceptable scientific standard, however I have significant reservations, as outlined above.

# Response

**Published:** 2022-07-10

**Authors:** marta nunes

**Affiliations:** University of the Witwatersrand, South Africa

Nunes et al. show results indicating that PBMCs collected from pre-pandemic donors respond to SARS-CoV-2 viral protein antigens, indicating that a certain pool of the South African population has resistance to this virus. The authors point out that this may be caused by a cross-reactivity induced by infections with the common cold coronaviruses, which previously was suggested by the others.

 Major points:

 1.           The authors used very high concentrations of peptide pools (1-2 mg/ml) while others used concentrations up to 1μg/ml. Please explain this. Did the authors perform any preliminary experiments to choose the optimal concentrations?

Authors: We are extremely sorry but there was a typo in the concentrations, as the reviewer pointed out the concentrations are mg/ml ( micro grams). This has been corrected now.

 2.           Table 2. It is completely incomprehensible, what do the values in the brackets mean e.g. (6.2, 10.3)? The authors should present their results in a comprehensible and clear way. This is not the case here.

Authors: We believe that the confusion is from the type of formatting used. We have now tried to make it more clear that the table is divided in 3 parts: 1) Responses to Interferon g, assessed by: i) SFCs per 10^6 PBMCs geometric mean and corresponding 95% confidence interval, and ii) count and percentage of responders; 2) Responses to Interleukin 2, assessed by: i) SFCs per 10^6 PBMCs geometric mean and corresponding 95% confidence interval, and ii) count and percentage of responders; 3) Count and percentage of responders to either Interferon g or Interleukin 2.

 The 2 values in brackets represent the 95% confidence interval of the geometric means as stated in the 1 ^st^ row of those sections.

 3.           The discussion is very cursory, and I believe the authors should put more emphasis on it. First, more articles that present data from unexposed donors responding to SARS-CoV-2 proteins should be cited and discussed. The authors should consider the vaccination status of the donors tested, as it is known that not only past infections with common cold coronaviruses but also vaccinations (with e.g. BCG or pneumococcal) can affect the immune system's response to this virus.

Authors: The discussion has been expanded, references added and the issue of heterologous vaccinations is now cited as a limitation.

 4.           The authors explain the response of some unexposed donors to SARS-CoV-2 by the cross-reactivity induced by common cold coronavirus infections. It is known that these infections are seasonal. The authors should present what the climate is in South Africa; when it most often comes to common colds; and what the scale is (how many people are infected with common cold coronaviruses).

Authors: data on the burden of common cold coronaviruses among South African adults has not been described. Although these viruses are normally seasonal, we are not aware of any in-depth study that looked at the annual circulation of these viruses in South Africa.

 5.           Please reveal statistical tests that have been used to analyze results.

Authors: please see reply to comment 2 of reviewer 1.

# Peer review: Reviewer response for version 1

**DOI:** 10.21956/gatesopenres.14618.r31832

**Published:** 2022-03-16

**Authors:** Helen Wagstaffe

**Affiliations:** 1 Imperial College London, London, UK

The article by Nunes *et al.* is a well written report of a pilot study conducted to measure T-cell responses to SARS-CoV-2 in unexposed individuals. The study utilises samples collected pre-pandemic and COVID-19 samples collected during the pandemic as positive controls. The study methods use the dual-colour FlouroSpot to measure both IFN-g and IL-2 producing T cells highlighting 2 different functions of T cells. The article concludes the presence of cross-reactive T cells in  samples collected pre-pandemic from South African women and speculates this may be due to past exposure to common cold coronaviruses. 

 In Table 1, please make clear what the round bracket and square bracket numbers are, especially for CD4+ cell count and HIV viral load.

 In the results section starting 'Interleukin-2', please provide the p-value for comparison between non-pregnant and pregnant women as for other significant comparisons. Please also state the kind of statistical test used for this comparison and any multiple comparison correction used.

 In the discussion, the authors suggest the highest level of cross-reactivity is in non-structural epitopes, recent work consistent with this includes Swadling *et al.* (2022 ^1^) and Kundu *et al.* (2022 ^2^).

Is the work clearly and accurately presented and does it cite the current literature?

Partly

If applicable, is the statistical analysis and its interpretation appropriate?

Yes

Are all the source data underlying the results available to ensure full reproducibility?

Yes

Is the study design appropriate and is the work technically sound?

Yes

Are the conclusions drawn adequately supported by the results?

Yes

Are sufficient details of methods and analysis provided to allow replication by others?

Yes

Reviewer Expertise:

Infectious disease immunology.

I confirm that I have read this submission and believe that I have an appropriate level of expertise to confirm that it is of an acceptable scientific standard.

## References

[1] : Pre-existing polymerase-specific T cells expand in abortive seronegative SARS-CoV-2. *Nature* .601(7891) : 10.1038/s41586-021-04186-8 110-117 10.1038/s41586-021-04186-8 34758478PMC8732273

[2] : Cross-reactive memory T cells associate with protection against SARS-CoV-2 infection in COVID-19 contacts. *Nature Communications* .2022;13(1) : 10.1038/s41467-021-27674-x 10.1038/s41467-021-27674-x PMC874888035013199

# Response

**Published:** 2022-07-10

**Authors:** marta nunes

**Affiliations:** University of the Witwatersrand, South Africa

The article by Nunes et al. is a well written report of a pilot study conducted to measure T-cell responses to SARS-CoV-2 in unexposed individuals. The study utilises samples collected pre-pandemic and COVID-19 samples collected during the pandemic as positive controls. The study methods use the dual-colour FlouroSpot to measure both IFN-g and IL-2 producing T cells highlighting 2 different functions of T cells. The article concludes the presence of cross-reactive T cells in  samples collected pre-pandemic from South African women and speculates this may be due to past exposure to common cold coronaviruses.

Authors: We thank the Reviewer.

 In Table 1, please make clear what the round bracket and square bracket numbers are, especially for CD4+ cell count and HIV viral load.

Authors: We have now included in the footnote that “Numbers in SQUARE brackets represent the number of participants with available information.”

 In the results section starting 'Interleukin-2', please provide the p-value for comparison between non-pregnant and pregnant women as for other significant comparisons. Please also state the kind of statistical test used for this comparison and any multiple comparison correction used.

Authors: p-value has now been included for the requested comparison. In the Analysis section of the Methods we have now added that “Geometric mean number of SFC/106 PBMCs and the corresponding 95% confidence interval (95%CI) were estimated using logarithmic transformation and compared between study cohorts by Student’s t-test. Responders were defined as individuals with ≥20 SFCs/106 PBMCs after subtraction of the SFCs in unstimulated control wells and with concomitant ≥2-fold increase over the unstimulated wells, and the proportion of responders were compared by Chi-square or Fisher’s exact-tests.”

 In the discussion, the authors suggest the highest level of cross-reactivity is in non-structural epitopes, recent work consistent with this includes Swadling et al. (20221) and Kundu et al. (20222).

Authors: The 2 suggested references have been added.
